# Do Social Networks Influence Small-Scale Fishermen’s Enforcement of Sea Tenure?

**DOI:** 10.1371/journal.pone.0121431

**Published:** 2015-03-30

**Authors:** Kara Stevens, Kenneth A. Frank, Daniel B. Kramer

**Affiliations:** 1 Michigan State University, Department of Fisheries and Wildlife, East Lansing, Michigan, United States of America; 2 Michigan State University, Department of Counseling, Educational Psychology and Special Education, East Lansing, Michigan, United States of America; 3 Michigan State University, James Madison College, East Lansing, Michigan, United States of America; Leibniz Center for Tropical Marine Ecology, GERMANY

## Abstract

Resource systems with enforced rules and strong monitoring systems typically have more predictable resource abundance, which can confer economic and social benefits to local communities. Co-management regimes demonstrate better social and ecological outcomes, but require an active role by community members in management activities, such as monitoring and enforcement. Previous work has emphasized understanding what makes fishermen comply with rules. This research takes a different approach to understand what influences an individual to enforce rules, particularly sea tenure. We conducted interviews and used multiple regression and Akaike’s Information Criteria model selection to evaluate the effect of social networks, food security, recent catch success, fisherman’s age and personal gear investment on individual’s enforcement of sea tenure. We found that fishermen’s enforcement of sea tenure declined between the two time periods measured and that social networks, age, food security, and changes in gear investment explained enforcement behavior across three different communities on Nicaragua’s Atlantic Coast, an area undergoing rapid globalization.

## Introduction

Fisheries are a challenging system to manage due to the mobility and nonexcludability of the resource. Evidence from around the world has demonstrated the effectiveness and conservation benefits of a variety of sea tenure regimes [1],[2]. Not surprisingly, fisheries with enforced rules result in greater fish biomass and abundance, improved habitat and increased fishermen’s incomes [3],[4]. Evidence suggests that fisheries have better ecological, social and economic outcomes under a co-management regime in which responsibility for management is shared between resource users and typically, a government agency [5],[6],[7],[8]. In addition, co-management implies local involvement in rule-making and enforcement, which reduces transaction costs for enforcement [9],[10].

Small-scale fisheries in developing countries are often remote and outside the reach and influence of central governments, but this does not presume a lack of governance. Community institutions, be they formal or informal, hold the responsibility and burden to ensure rule compliance and handle conflict [11],[12]. Successfully managed common pool resources depend on the ability of users to undertake enforcement themselves [13],[12]. This reliance on enforcement from resource users is especially true in small-scale fisheries of the remote developing world. The commons literature provides rich evidence of self-enforcing communities. A classic example from Maine demonstrates a strong social norm of territoriality, enforced by fishermen themselves with the ability to organize and enforce in the absence of a government-recognized legal framework [14]. Other studies suggest that community characteristics such as high degrees of social capital, clear rules and sanctions, the involvement of resource users in establishing regulations and cross-scale linkages between communities and higher levels of governance improve enforcement at the community level [15],[16],[17].

It is important to distinguish between compliance and enforcement, particularly in management of the commons. Enforcement is one aspect that contributes to overall compliance, yet when we think of individual behavior, the act of enforcing rules requires an elevated sense of commitment to community-based governance compared to simply complying with rules. In the case of illegal fishing, while a fisherman may limit his fishing to the legal grounds (compliance), he may not go so far as to prevent and confront others from fishing in restricted areas (enforcement). Enforcement has been examined from the top-down by exploring the question of what makes people comply with rules [18],[19],[20],[21],[22],[23] or the effectiveness of government agencies in using enforcement to achieve compliance [24]. Peer group solidarity, moral motives and the legislator’s authority have been found to explain fishermen’s compliance [25],[26],[27],[28]. Studies have also found a fisherman’s motivation to comply varies depending on the degree to which he violates rules [25]; similarly there is variation in fishermen’s willingness to denounce others for illegal fishing [29]. Yet there have been few empirical studies exploring the motivation of individual resource users to participate in enforcing restrictions on illegal fishing activities amongst themselves, a fundamental component of co-management regimes.

One pathway by which social pressure is applied is the social network, in other words, the relationships amongst individuals in a defined population such as a community or group of resource users. Networks have been analyzed at multiple scales to understand behavioral outcomes in health, governance, education and business [30],[31],[32], and more recently social network analysis has been applied to explain dynamics in agriculture and fisheries management. Social network characteristics such as the level of network cohesion or the existence of bridging ties can affect cooperation on resource management issues, conflict resolution, influence on decision-making processes, information sharing and community-based monitoring and enforcement of rules [33],[34],[35],[36]. Social network structure can also change as a result of changes in resource condition [37]. Other network studies identify links between individual network position, such as centrality, or personal network size, and farmers’ ability to accept agricultural extension information or plant diverse species [38],[39]. In this study, rather than trying to understand how an individual’s structural position in a network affects decision-making, we study explicitly how his ties affect his behavior. That is, we examine how the behavioral characteristics of a fisherman’s social network affect his own behavior, particularly his enforcement of sea tenure (i.e. enforcement behavior). While Kuperan & Sutinen [18] found that amongst other factors, social influence was important in determining fishermen’s compliance with rules, the question here is whether social ties influence a fisherman’s enforcement of rules.

We build on the framework of communal management of the commons to examine how individuals within a community experience norms in different ways, depending on the networks in which they are embedded [40]. Enforcement of social norms is a second-order free-rider problem in which the cost of punishing others is borne by a few, but benefits of the punishment are reaped by many [41]. This voluntary engagement in enforcement is supported by both theoretical and empirical studies [42],[43],[44]. Of particular relevance are studies of altruistic punishment, which find that group selection can play an important role in the motivation of an individual to expend costly resources to punish violators of social norms [45]. Social networks can provide the support for norm enforcement that is beneficial at the community level and contributes to fisheries sustainability. This study contributes to our understanding of social networks by examining how peer groups influence individual behavior while controlling for other factors such as prior behavior, food security, age, and investment in the fishery.

We explored this question in Nicaragua’s rapidly transforming Atlantic Coast region, which was connected via road to the country’s interior for the first time in 2007. Road connection has resulted in a host of direct and indirect changes to coastal communities, including expansion of markets for fisheries products, facilitated migration and the introduction of new technologies [46],[47]. Increased market access in these communities has resulted in a 40–65% increase in fish price (paid to fishermen) from 2010–2012 (pers. obs.). With widespread perceptions from local fishermen of declines in lagoon fisheries coupled with increased market value of fisheries products, we might expect enforcement of sea tenure to increase. In contrast, rapid large-scale changes and declining fish stocks may induce despair that the state of the resource is such that individual actions have no meaningful impact.

In this study, we examined 1) whether enforcement behavior amongst individual fishermen changed over time, 2) if social networks had an influence on that change and 3) other factors that explain changes in fishermen’s behavior.

## Study Site

**Fig 1:**
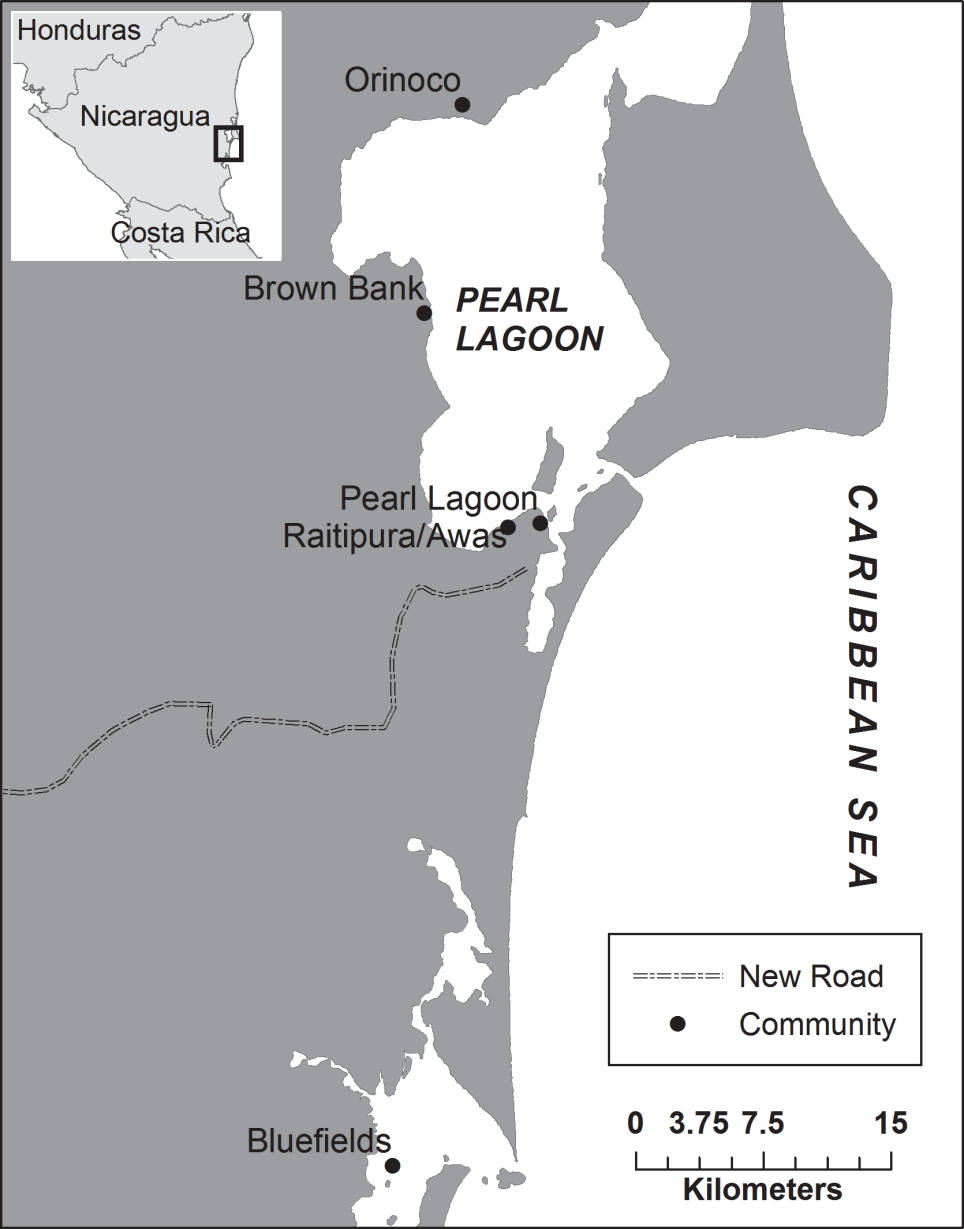
Nicaragua’s Atlantic Coast. The capital of Nicaragua’s Southern Autonomous Region is Bluefields. The study took place in the municipal capital north of Bluefields, Pearl Lagoon.

Pearl Lagoon refers to a municipality, a community and an estuary. For the purposes of clarity and to be consistent with previous work, we refer to the estuary as the Lagoon, the municipality as the Municipality and the community as Pearl Lagoon. Nicaragua’s Southern Autonomous Region (RAAS) is divided into thirteen municipalities. The Pearl Lagoon municipality prohibits artisanal fishermen and semi-industrial boats from other municipalities from fishing in its waters [48]. The four communities studied in this research, Raitipura, Awas, Brown Bank and Orinoco, vary in distance from the main town of Pearl Lagoon, which is one of the closest points of entry from the municipality to the south, Bluefields (Fig 1). Bluefields is also situated on an estuary and is located approximately 40 km from Pearl Lagoon, which is reached through freshwater canals and rivers. While both municipal centers lie on a lagoon, the Bluefields Bay is less than half the surface area of the lagoon and supports a human population 10 times the size.

Most of the illegal fishing in the lagoon can be attributed to fishermen from Bluefields, who have been coming into the lagoon at least since 1997 [49], and possibly before, the cause of which may be linked to an exhaustion of commercially viable fishery resources in Bluefields Bay. Lagoon fishermen report that Bluefields fishermen violate several local social norms (K. Stevens, qualitative interview data, pers. obs.). In addition to reports of illegal fishing, Bluefields interlopers are reported to use gill nets with a mesh size smaller (3-inch) than the minimum acceptable size (4-inch) and use 3–4 times the number of gill nets typically used by most local fishermen to ensure profitability (X. Gordon, pers. comm. K.Stevens, qualitative interview data). Additionally, they carry multiple iceboxes capable of carrying several hundred pounds each, often attempt to enter and exit the lagoon under cover of night, and do not sell their product in the municipality, generating no tax revenue for the local government nor income for local fish-buying middlemen (X. Gordon, pers. comm.). However, they do sometimes invite local fishermen to work on their boats in an attempt to legitimize their activities (K.Stevens, qualitative interview data). Based on interviews, fishermen may report the outsiders whose activities do not align with established norms to their communal board or the municipal authorities (K.Stevens, qualitative interview data, pers. obs, X. Gordon, pers. comm.). Lacking resources to make regular patrols, the municipal authority only expends resources for on-water patrol if they hear complaints from fishermen about illegal fishing (X. Gordon, pers. comm.).

## Methods

Interviews were conducted with fishermen in four communities in February of 2011 and 2012 (n = 285 in each year). The same fishermen were interviewed in both years. They ranged in age from 18–71. A core group of fishermen was identified based on pilot interviews in 2010. Thereafter, a snowball interview method was used to define the population of fishermen based on each person’s responses to the following questions regarding their social networks: 1) with whom do you fish and 2) with whom are you friends? Interviews were conducted with all fishermen mentioned in response to these questions until saturation was reached. Fishermen were also asked whether they had seen or verbally confronted a fisherman violating sea tenure in the past year, the frequency of confrontation(s), whether it led to physical violence and whether they requested action or support from government entities, such as the communal board, communal leader or local municipality (S4 Table). This is considered an exhaustive list of likely enforcement actions. An enforcement score was tabulated based on individual responses to these questions, such that one point was given for each enforcement action taken. The lower the score, the fewer actions the fisherman undertook in confronting or reporting outsiders fishing in the lagoon. The responses were standardized between years to only compare enforcement of sea tenure between those who had/had not encountered outsiders in both years measured.

Trained community data collectors interviewed fishermen in each community on a weekly basis about their fishing trips from March 2010—June 2012. They collected data on type, size and number of gear used, fishing location, duration of fishing trip, number and total pounds of each species captured and the price per pound for species sold. These data were used to calculate the covariate, *income*, described in the model statements below.

The Institutional Review Board of Michigan State University approved these studies. Written consent was obtained from participants prior to the start of the survey. The consent form, which included the purpose of the study, who would have access to the data, how the data would be used and the right to refuse was explained to participants. Thereafter participants signed the consent form.

### Data Analysis

To examine whether individual enforcement behavior changed between the two years, we analyzed individual differences in enforcement action in four communities using a paired t-test (R v. 2.15.1, 2012).

Two social network measures were used in our analysis. First, we used KliqueFinder to examine social network data and identify possible subgroups in each community [50], [51]. KliqueFinder is one of several programs capable of identifying subgroups in network data. It does so by identifying which individuals are more likely to interact with each other than with others in the community by iteratively maximizing the odds ratio of ties between fishermen and their subgroup membership [50]. Subgroups have been shown to have a strong influence on behavior in other contexts [52] and are important in assessing outcomes in resource governance [35]. Second, we determined each fisherman’s egocentric network, those individuals directly identified by the fisherman as fishing partners or friends. Awas is a small community that was established due to land shortages in Raitipura and the two are in close proximity and linked by family relations and intermarriage. Because fishermen from Awas have fishing partners and friends from Raitipura and vice versa, the two communities were analyzed separately and together.

We used Akaike’s Information Criteria corrected for small sample size (AICc) to evaluate twelve candidate models’ ability to explain fishermen’s enforcement behavior [53]. These candidate models were selected a priori based on the inclusion of economic and ecological explanatory variables, social network influence variables, a combination of the two and the global model. We included fishermen’s income as a variable given the positive correlation between resource abundance and enforcement, as well as other evidence indicating stock crashes induce a stronger conservation ethic and support for established tenure rights [54],[3],[55]. Age has been shown to be positively associated with rule compliance and we expect that it could also be a factor affecting enforcement behavior [56],[57]. While studies have examined how fisheries abundance and diversity affect food security of coastal communities [58],[59], the reverse pathway, how food security affects a fisherman’s harvest practices, has been rarely studied. Yet we know from other disciplines that food insecurity has profound effects on health and physiology, which can influence behavior [60],[61]. We included gear investment as a proxy for a fishermen’s investment in the fishery based on the logic that those with greater investment have greater incentives to engage in management of the fishery [62]. The model with the lowest AICc score was considered the most likely [53]. In addition, we report the difference in AICc scores between the best-fit model and other candidate models (ΔAICc). Delta AICc values of 0 indicate the model with the most explanatory power; and models with ΔAICc values less than two are considered equally likely to be the best model [53]. The weights (w_i_) sum to one and indicate how much support that model has amongst candidate models in explaining the outcome.

The fishermen’s enforcement score in the second year was the dependent variable, and the effect of the egocentric network, the subgroup, the fishermen’s prior behavior and other economic and ecological factors were explanatory variables (*Eq. 1*).

Yit=ρ1∑i'xii'*yi't−1/∑i'xii'+ρ2priorbehaviorit−1+ρ3ageit+ρ4foodsecurityit+ρ5geari+ρ6incomeit+ρ7groupit−1 Eq. 1

In this model, *Y*_*it*_ is the enforcement behavior of fisherman *i* at time *t*. The relationship between fisherman *i* and his friends/fishing partners *i’* is described as *x*_*ii’*_. *y*_*i’t-1*_ is the prior behavior of fisherman *i’*. Thus the egocentric network exposure term (referred to as *ego* in model statements) $\sum_{i'}x_{ii'}*y_{i't-1}/\sum_{i'}x_{ii'}$ represents the exposure to the practices in one’s network, in other words, the mean enforcement score in year one amongst the fishermen’s selected friends and fishing partners [63]. The mean exposure to members of the fishermen’s friends or fishing partners subgroup as identified by KliqueFinder is encompassed in the term *group*. The first year enforcement measure in the model statement is described as the *prior behavior* of fisherman *i*. Age of the fishermen was measured in year two (*age*). Food security was measured directly in one community, Orinoco. It was based on interviewee’s responses to four questions about food scarcity, affordability and uncertainty based on an adaptation of the United States Department of Agriculture Short Form Food Security Survey [64] (S2 Table). Without direct measures of food security in Raitipura and Awas, we divided household wealth by household size to generate a metric of wealth per household member, which serves as a proxy for food security (*food* in model statements). We have both of these metrics in 11 households in Orinoco. The two measures are significantly correlated based on Pearson’s correlation test (r = .63, p-value = .04). Principal components analysis (R v.2.15.1, 2002, princomp package) was used to generate a household wealth measure from binary responses to ownership of household goods [65]. Fishermen’s personal gear investment was estimated based on the difference in the summed monetary value of all fishing gear owned between the first and second year including boat, motor, dugout canoe and various types of gear like gill net, cast net and trawl net (*gear*) (S3 Table). The term, *income*, is a measure of the average income per trip earned by the individual fishermen in the two months prior to the year two behavior measure. It includes shrimp and fish harvest with any gear type including handline, cast net and gill net. In order to approximate the near-term individual economic status of fishermen the measure is not species or gear specific. Data on price per pound was recorded for each species and total income per trip was summed across all species. Most commonly captured fish species in this metric include mojarra *Eugerres plumieri*, catfish *Bagre marinus*, snook *Centropomus* spp., and croaker *Micropogonias furnieri*. This paper includes explanatory variables that are contextually important and highly variable in small-scale fisheries. In addition to the influence of their social network, we might expect that fishermen with a greater investment in the fishery (*gear*), whose recent income was low (*income*), who have more experience (*age*), and/or who are food insecure (*food*) to be more likely to enforce sea tenure to remove illegal fishermen.

We tested for multicollinearity by evaluating variance inflation factors of explanatory variables; all had values between 1 and 2 (R v. 2.15.1, 2012). Raitipura and Raitipura/Awas friends and partners models met the assumptions of regression without transformation. The dependent variable in the Orinoco friends and partners models was square-root transformed to meet assumptions of multiple linear regression.

There are concerns about potential dependencies in estimating any social network model (e.g., [66],[67]). Estimated influence is biased if the errors are not independent of the network exposure term (see [68], equations 1.2–1.4). The estimate of influence will be positively biased if there is some unexplained aspect of enforcement behavior that is related to the network exposure. The most compelling source of such dependencies would be if people choose to interact with others whose behaviors are similar to their own, known as selection in the network literature [69]. Those who engaged in enforcement in the first year might have chosen to interact with similar others between the first and second year, and also would have been inclined to engage in enforcement behaviors in the second year. Because the network exposure term is likely confounded with prior enforcement behavior, the model used here includes a control for prior enforcement behavior.

A second concern would arise if the model of a fisherman’s behavior was a function of the contemporaneous behavior of those in his network. This would essentially put the outcome on both sides of the model in which case the errors would be directly related to the exposure term. It is for this reason that we model enforcement behavior as a function of the previous behaviors of others in one’s network. This avoids creating dependencies between the errors and predictors by putting the same variables on both sides of the model.

## Results

### Behavior change

Brown Bank was the only community in which enforcement scores between the two years were not significantly different (Table 1). In all communities, the enforcement score decreased in the second time period, indicating fewer mean enforcement actions. Raitipura had the highest enforcement score of all communities. Enforcement scores decreased with increasing distance from the municipal center and the newly constructed road.

**Table 1:** Results of t-test of change in individual enforcement actions between 2011 and 2012 in four communities of the Pearl Lagoon basin.

	**2011**		**2012**			
	**Mean**	**SE**	**Mean**	**SE**	**df**	**p**
**Brown Bank**	3.09	.60	2.53	.61	16	.31
**Orinoco**	1.72	.19	.79	.12	102	0.00*
**Raitipura**	3.47	.33	2.58	.28	73	.002*
**Raitipura/Awas**	3.22	.28	2.49	.24	90	.005*

*p<.01.

When we compare specific enforcement actions of fishermen who observed outsiders in both years, we see fewer direct confrontations with outsiders amongst Orinoco and Brown Bank fishermen, but more in Raitipura and Awas (Table 2). From 2011 to 2012, across all communities there were fewer (or no change in) requests to the community leader and community board for assistance in preventing illegal fishing, and fewer requests to the municipality. Of 96 direct confrontations in 2011, one resulted in physical attack or threat of attack. Of 65 direct confrontations in 2012, zero resulted in physical attack. Across all fishermen interviewed in 2011, 25% of fishermen who observed illegal fishing by those from outside the municipality took no enforcement action. In 2012, this had increased to 42%. We used Cronbach alpha as a measure of internal consistency to assess the degree to which the enforcement questions measured enforcement. In 2011, the Cronbach alpha was. 73 and in 2012 it was. 69, which was calculated from questions about reporting to the communal leader, communal board and municipal staff since those questions were asked consistently to all respondents regardless of observation of illegal activity (Table 2). Cronbach’s alpha coefficients showed good internal consistency in both years.

**Table 2:** Percent change in specific individual enforcement actions from 2011–2012.

	**Observation of illegal fishers**	**Confrontation with illegal fishers**	**Report to communal leader**	**Report to communal board**	**Report to municipality**
**Brown Bank**	-9%	-4%	0	-50%	-17%
**Orinoco**	-15%	-56%	-37%	-47%	-63%
**Raitipura**	-15%	10%	-11%	-17%	-3%
**Raitipura/Awas**	-20%	22%	-5%	-12%	-9%

Cronbach alpha_2011_ = .73; Cronbach alpha_2012_ = .69.

### Social Network

Based on KliqueFinder results, we determined that between 4 and 24 distinct subgroups of friends and fishing partners exist within the communities, depending on the size of the fishermen population (Table 3). When analyzed independently, Awas did not show evidence of distinct subgroups. Across all communities and both networks, the mean size of the subgroup was 4.7 individuals; the mean size of the egocentric network was 3.0.

**Table 3:** Results of KliqueFinder analysis of social network data showing evidence of distinct subgroups in friends and fishing partners networks in four communities of the Pearl Lagoon basin.

**Community**	**Friends subgroups**	**Fishing partners subgroups**
**Awas** (n = 23)	None	None
**Brown Bank** (n = 31)	4^*^	4^*^
**Orinoco** (n = 130)	24^*^	20^*^
**Raitipura** (n = 92)	18^*^	16^*^
**Raitipura/Awas** (n = 115)	22^*^	22^*^

*p<.01.

### Factors influencing behavior change

With year two enforcement behavior as the dependent variable, 12 candidate models were evaluated. We first ran these models for the friends network (Table 4). In Orinoco, models H, I, J and K were considered highly likely and differed in the inclusion of age and egocentric or subgroup network terms. Gear investment and income were also included in the best-fit models in Orinoco. In both Raitipura and Raitipura/Awas, the global model was highly supported (w_i_ = 1.0). Sample size in Brown Bank was too small to effectively evaluate the models. Next, we ran the same twelve candidate models to examine the effects of the fishing partners social network (Table 5, See S1 Table for global model fit characteristics). In Orinoco, Raitipura and Raitipura/Awas the same 4 models selected in the previous analysis were again selected.

**Table 4:** Model selection results of the influence of a fisherman’s friends network on enforcement behavior. ^1^

**Models**		**Orinoco**			**Raitipura**			**Raitipura/Awas**		
	**k**	**AICc score**	Δ**AICc**	**w**_i_	**AICc score**	Δ**AICc**	**w**_i_	**AICc score**	Δ**AICc**	**w**_i_
*Ecological and Economic Models*										
^A)^ age + gear + income + food	4	187.1	26.9	0	312.8	36.2	0	325.2	41.1	0
^B)^ age + gear + income	3	204.7	44.5	0	333.9	57.3	0	337.8	53.7	0
^C)^ gear + income	2	204.3	44.1	0	344.6	68.0	0	343.9	59.8	0
*Network Influence Models*										
^D)^ ego + prior behavior	2	202.3	42.1	0	309.1	32.5	0	371.1	87	0
^E)^ ego + group + prior behavior	3	203.6	43.4	0	310.6	34.0	0	354.8	70.7	0
^F)^ group + prior behavior	2	201.4	41.2	0	308.6	32.0	0	352.6	68.5	0
*Prior Behavior*										
^G)^ prior behavior	1	200.2	40.0	0	307.9	31.3	0	369.1	85	0
*Combined Models*										
^H)^ gear + income + ego + prior behavior	4	160.5	0.3	.23	304.2	27.6	0	305.3	21.2	0
^I)^ gear + income + group + prior behavior	4	160.7	0.5	.21	303.9	27.3	0	303.7	19.6	0
^J)^ age + gear + income + ego + prior behavior	5	160.2	0	**.27**	294.3	17.7	0	297.9	13.8	0
^K)^ age + gear + income + group + prior behavior	5	160.7	0.5	.21	293.9	17.3	0	296.4	12.3	0
*Global Model*										
^L)^ ego + group + prior behavior + income + age + gear + food	7	162.9	2.7	.07	276.6	0	**1**	284.1	0	**1**

^1^ See S1 Table for parameter estimates and adjusted r-squared for models of best fit.

**Table 5:** Model selection results of the influence of a fisherman’s fishing partners network on enforcement behavior.^1^

**Models**		**Orinoco**			**Raitipura**			**Raitipura/Awas**		
	**k**	**AICc Score**	Δ**AICc**	**W**_i_	**AICc Score**	Δ**AICc**	**W**_i_	**AICc Score**	Δ**AICc**	**W**_i_
*Ecological and Economic Models*										
^A)^ age + gear + income + food	4	187.1	26.6	0	312.8	37.2	0	325.2	39.4	0
^B)^ age + gear + income	3	204.7	44.2	0	333.9	58.3	0	337.8	52.0	0
^C)^ gear + income	2	204.3	43.8	0	344.6	69.0	0	343.9	58.1	0
*Social Network Models*										
^D)^ ego + prior behavior	2	202.4	41.9	0	310.1	34.5	0	371.3	85.5	0
^E)^ ego + group + prior behavior	3	204.3	43.8	0	312.0	36.4	0	371.3	85.5	0
^F)^ group + prior behavior	2	202.1	41.6	0	309.7	34.1	0	369.5	83.7	0
*Prior Behavior*										
^G)^ prior behavior	1	200.2	39.7	0	307.9	32.3	0	369.1	83.3	0
*Combined Models*										
^H)^ gear + income + ego + prior behavior	4	160.6	0.1	.24	304.6	29.0	0	306.1	20.3	0
^I)^ gear + income + group + prior behavior	4	160.8	0.3	.22	304.5	28.9	0	303.9	18.1	0
^J)^ age + gear + income + ego + prior behavior	5	160.5	0	**.25**	294.3	18.7	0	297.9	12.1	0
^K)^ age + gear + income + group + prior behavior	5	160.7	0.2	.23	294.2	18.6	0	297.1	11.3	0
*Global Model*										
^L)^ ego + group + prior behavior + income + age + gear + food	7	163.1	2.6	.07	275.6	0	**1**	285.8	0	**1**

^1^ See S1 Table for parameter estimates and adjusted r-squared for models of best fit.

**Fig 2:**
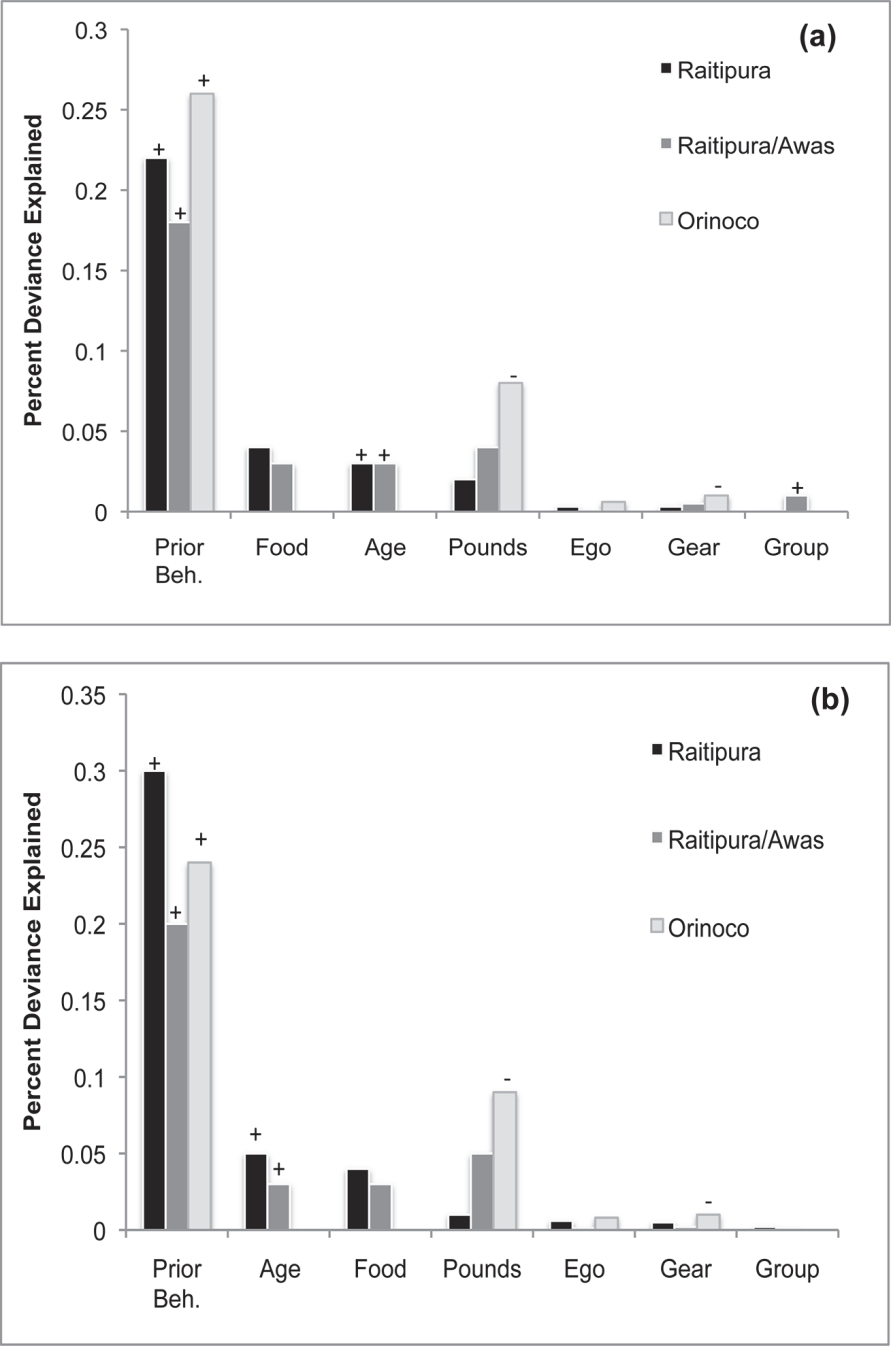
Percent deviance of each parameter. Percent deviance explained by each parameter in the best-fit model explaining fishermen’s enforcement behavior for the (a) friends network and the (b) fishing partners network. If the parameter estimate plus/minus the standard error does not include zero, the direction of the parameter estimate is indicated above the column.

We calculated the percent deviance contributed by each variable in the model of best fit for each community (Fig 2). If the parameter estimate plus or minus standard error did not include zero, the sign is given for the explanatory variable. In both friends and fishing partners network models, in all communities, prior behavior explained most of the deviance. Thereafter, we find fishermen’s age, food security, recent catch (*income*), and social network measures to explain deviance in enforcement of sea tenure. The relative importance of these factors varies by community.

## Discussion

This study explores why fishermen enforce restrictions on illegal activity, particularly violations of sea tenure, and finds that accounting for a fisherman’s social network, food security, age, gear investment and income is important in predicting enforcement actions. Social network terms were included in models of best-fit across all communities, consistent with the normative model of behavior that fishermen are influenced by social norms and their peer group, though networks appear to play a minor role relative to other factors in the model [19].

We compared the influence of two types of networks—friends and fishing partners—on enforcement actions and found little difference between the two. The questions we used to measure enforcement asked about both on-water and on-land enforcement actions. It is logical that both social networks that were measured influence this behavior since on-water confrontation with an illegal fisherman would likely be accomplished with a fishing partner, while follow-up with local or municipal government would potentially be accomplished with friends. Based on qualitative responses during interviews, there is community variation in how to handle outsiders fishing in Pearl Lagoon. While some fishermen expressed staunch opposition to outsiders fishing in the lagoon on any terms, some allow it depending on the methods being used or based on the belief that all people have a right to make a living, and in some cases local fishermen join the illegal fishing group. Few fishermen joined illegal fishermen, depending on the community, and for various reasons these individuals were not included in the models. The lack of a widely held and agreed upon social norm related to sea tenure within the communities may affect the influence of a fisherman’s social network. Sanctioning those who violate norms can be a socially costly action, though less so when the violation is commonly practiced within a community [70]. Social networks can have both a positive and negative effect on individual enforcement. A positive effect can be interpreted as the more an individual’s network members engage in enforcement behavior, the more likely that individual is to engage in the behavior. A negative effect means that the more an individual’s network members engage in enforcement, the *less* likely that individual is to engage in the behavior. This situation can arise when the behavior is regarded as less desirable or in cases of prevalent free-riding. For example, in communities where network influence of enforcement is negative, there may be an over-reliance on certain individuals to enforce while others reap the benefits.

In all models with network influence terms we controlled for prior behavior, yet there was no evidence to support the model that only included prior behavior, indicating that behavior had changed and other factors explain the change. Further, the models that only included ecological and economic variables, such as food security, age, gear investment and income received no support in explaining enforcement behavior.

The lack of enforcement of sea tenure by the majority of fishermen in the lagoon coupled with the variation in response to illegal fishers may explain why network terms did not explain most of the deviance in the outcome variable relative to other variables in the model. In addition to the influence terms, this study found that other factors also affect fishermen’s enforcement behavior. In all communities, gear investment, income and age explained deviation in enforcement of sea tenure. Gear investment was negatively related to enforcement in Orinoco, a counterintuitive result as it would be expected that fishermen heavily invested in the fishery would also be invested in protecting it. Individuals with lots of gear often rent it out and thus may be a step removed from management of the fishery and lack knowledge about threats to the resource. Gear lenders may also demonstrate little knowledge of declining fisheries [71]. Age had a positive effect on enforcement actions in Raitipura and Awas, implying the relevance of engagement with communal elders to increase community involvement in enforcement. This is consistent with the compliance literature that finds rule compliance is positively related to age [56],[57]. In Raitipura and Awas, food security also explained fishermen’s enforcement actions, but in Orinoco this factor was not included in models of best fit. Understanding the contribution of small-scale fisheries to food security is of increasing importance [72],[73], particularly if food security affects fishermen’s management behavior as suggested in this study. Decreases in food security as a result of declining fisheries have the potential for a negative or positive feedback to the fishery depending on fishermen’s response to these changes. If food-insecure fishermen choose not to expend resources to prevent illegal fishing, the long-term result may be further deterioration of food security. In Raitipura/Awas we see a positive relationship between food security and enforcement suggesting that as fishermen are more food secure they are likely to enforce. One data limitation is that we do not know precisely the contribution of fish to food security, though it is a substantial portion of the diet (pers. obs.).

Enforcement of sea tenure by fishermen decreased amongst all communities from 2011–2012. While at least 80% of fishermen in each community had witnessed outsiders fishing in the lagoon in the year preceding 2011, the following year this percentage had decreased to 67–75%, depending on the community. This does not explain the decline in enforcement score between years since we standardized data to only compare enforcement of sea tenure between those who had or had not encountered outsiders in both years measured. Physical violence did not appear to be a relevant factor preventing fishermen from enforcing sea tenure. There was one incident reported in 2011 from Raitipura in which an illegal fisher threatened a local fisherman with a harpoon. In other communities there were zero reports in both years. The lack of evidence for confrontations that end in violence suggests that the risk of physical harm does not play a role in enforcement behavior. An abundance of Bluefields fishermen in the first year coupled with active enforcement via direct confrontation and regular reports to the municipal fisheries inspector may have resulted in a ‘cooling effect’ in which fewer Bluefields fishermen came to the region in the second year. In addition, there was a significant seizure of Bluefields fishermen’s gear, thermos and fish by the Pearl Lagoon municipal fisheries inspector in the second year, an enforcement measure widely noticed locally for its severity since previous enforcement measures by the municipality only consisted of verbal warnings (pers. obs., X. Gordon, pers. comm.). The unintended side effect of municipal enforcement of sea tenure in the lagoon may have been a resulting reliance on the municipality to handle illegal fishermen [3]. In addition, anecdotal reports from interviews conducted as part of this survey suggest that the harvest practices of the Bluefields fishermen in the lagoon were generally less offensive to local fishermen in the second year. Fishermen, particularly in Raitipura and Awas, indicated that some Bluefields fishermen were fishing in alignment with local norms and several fishermen reported that they do not confront outsiders as long as their methods are consistent with how locals fish. This is contrasted with first year anecdotal reports of many Bluefields fishermen “abusing” the lagoon by using small-mesh gill nets. These two distinct approaches to illegal fishers are essentially based on whether the fisherman is fishing to eat or fishing for substantial profit, and the fishermen of the lagoon are more sympathetic to the former, a distinction also observed in a Norwegian fishery [21]. In any case, the apparent reduction in fishermen’s enforcement of sea tenure is not an encouraging indicator of sustainable fisheries management, which is difficult to achieve in a de facto open access rights regime [74]. It is unclear if the reduction in enforcement extends beyond sea tenure to other social norms designed for sustainable resource management. Of all the sanctions fishermen may choose to enforce, it is perhaps less risky and least costly to confront and sanction outsiders compared to fellow community members.

Mean community enforcement scores decreased between the two years with increasing distance from the municipal center and road terminus at the town of Pearl Lagoon. This could be explained by several factors. First, Raitipura and Awas have logistically easier and more frequent access to municipal staff, including the fisheries inspector. Second, the illegal fishermen concentrate their activities in parts of the lagoon more frequented by fishermen from Raitipura/Awas. With a higher likelihood of Raitipura fishermen encountering illegal fishermen and more competition for the resource, it is reasonable that they are more likely to enforce sea tenure. Finally, Raitipura and Awas are the only two Miskitu communities of the four studied in this research. Brown Bank is a predominantly Creole community, and Orinoco predominantly Garifuna. A recent study of Hawaii’s longline fishery found that ethnicity influences network structure, which may affect information flow, collaboration and overall management [75]. Given the history of the Atlantic Coast, Miskitu communities are in a better position to claim and assert their territorial rights to resources compared to other ethnic groups [76] (Nicaraguan Law 445).

There are multiple threats to the sustainability of the Pearl Lagoon fishery. Perhaps the regulation with the least local resistance to implementation is the prevention of illegal fishers from other municipalities from fishing in the lagoon. That it continues to be a problem after 15 years indicates that enforcement could be improved. While fishermen cannot be expected to stop all illegal fishing without support from the government, strengthening existing communal social norms of sea tenure and empowering fishermen to enforce their own rules and increase coordination with communal and municipal authorities can result in improved compliance and reduced costs for management [8].

Systems with monitors that are appointed by, accountable to, or are the resource users themselves is one of the eight design principles identified for successful institutions and empirically demonstrated to result in better management outcomes [77],[78]. Here we explore fishermen’s enforcement of sea tenure, yet understanding individual enforcement through the lens of social networks is relevant to the enforcement of any communal social norm. Fishermen are less likely to sanction others if there is no support amongst their social network for doing so. Just as public health officials use social networks to identify opinion leaders or “champions” in the community who are instrumental in driving behavior change related to certain health practices [79], fisheries managers could use knowledge of social networks to improve stakeholder communication, identify key people to engage in policy reform, and promote adoption of certain harvest practices. These types of ‘network interventions’ have been so far little used in natural resource management [79]. Additionally, if fishermen’s behaviors are affected by their network, analysis can uncover clustering of subgroups within communities each with distinctly evolving normative pressures, which can create challenges for management [35]. Using knowledge of social networks to strengthen traditional norms of resource use may result in improved effectiveness of co-management regimes, particularly during periods of social and ecological change that may be driven by rapid globalization.

## Supporting Information

S1 Table Parameter estimates (standard error) and model fit characteristics for the model of best fit in each community for both the friends and fishing partners’ social networks. (DOCX)

S2 Table Survey questions used to assess food security for fishermen adapted from USDA’s Six Item Short Form of the Food Security Survey (Interviewees responded with agree/disagree/neither agree nor disagree). (DOCX)

S3 Table Survey question used to assess gear ownership amongst fishermen in four communities around Pearl Lagoon, Nicaragua. (DOCX)

S4 Table Survey questions used to assess enforcement behavior amongst fishermen in four communities around Pearl Lagoon, Nicaragua. (DOCX)
