# Supplementary material for: Do Social Networks Influence Small-Scale Fishermen’s Enforcement of Sea Tenure?
Source: PLoS One. 2015 Mar 30;10(3):e0121431. doi: 10.1371/journal.pone.0121431 (PMC4379162; doi:10.1371/journal.pone.0121431)
Supplement: S1 Table — (DOCX) [file pone.0121431.s001.docx]

|  | **Friends** | | | **Fishing Partners** | | |
| --- | --- | --- | --- | --- | --- | --- |
|  | **Orinoco** | **Raitipura** | **Raitipura/ Awas** | **Orinoco** | **Raitipura** | **Raitipura/Awas** |
| **Ego** | -.04 (.06) | .04 (.13) | .0006 (.13) | -.02 (.05) | .14 (.14) | .04 (.15) |
| **group** | --- | -.11 (.23) | .30 (.22) | --- | -.22 (.25) | .10 (.24) |
| **Prior** | .23 (.04)** | .58 (.11)** | .46 (.09)** | .23 (.04)** | .58 (.10)** | .50 (.09)** |
| **income** | -.0001 (.0001) | -.0007 (.001) | -.0002 (.0007) | -.0001 (.0001) | -.0007 (.001) | -.0003 (.0007) |
| **Age** | .004 (.005) | .06 (.02)* | .06 (.02)* | .004 (.005) | .06 (.02)* | .06 (.02)* |
| **Gear** | -.00005 (.00003) | .0001 (.0002) | .0001 (.0001) | -.00005 (.00003) | .0001 (.0002) | .0001 (.0001) |
| **Food** | --- | .49 (.72) | .80 (.67) | --- | .61 (.71) | .69 (.67) |
| **P** | p<.001 | p<.001 | p<.001 | p<.001 | p<.001 | p<.001 |
| **r^2^ (adj.)** | .31 | .43 | .45 | .31 | .44 | .44 |

**S1 Table**. Parameter estimates (standard error) and model fit characteristics for the model of best fit in each community for both the friends and fishing partners’ social networks.

* p<.05; ** p<.001
