# Supplementary material for: Do Social Networks Influence Small-Scale Fishermen’s Enforcement of Sea Tenure?
Source: PLoS One. 2015 Mar 30;10(3):e0121431. doi: 10.1371/journal.pone.0121431 (PMC4379162; doi:10.1371/journal.pone.0121431)
Supplement: S2 Table — (DOCX) [file pone.0121431.s002.docx]

**S2 Table**. Survey questions used to assess food security for fishermen adapted from USDA’s Six Item Short Form of the Food Security Survey (Interviewees responded with agree/disagree/neither agree nor disagree).

| 1. Sometimes you can't afford to get all the food you need to feed your kids/family. |
| --- |
| 2. Sometimes you skip meals or eat less because there is not enough food. |
| 3. You worry about where your food will come from. |
| 4. You are losing weight or getting sick because you are not eating well. |
