# Supplementary material for: Do Social Networks Influence Small-Scale Fishermen’s Enforcement of Sea Tenure?
Source: PLoS One. 2015 Mar 30;10(3):e0121431. doi: 10.1371/journal.pone.0121431 (PMC4379162; doi:10.1371/journal.pone.0121431)
Supplement: S3 Table — (DOCX) [file pone.0121431.s003.docx]

**S3 Table**. Survey question used to assess gear ownership amongst fishermen in four communities around Pearl Lagoon, Nicaragua.

|  | 2. Do you own a _____? (Y/N) |
| --- | --- |
| Panga |  |
| Dorry (dugout canoe) |  |
| Motor |  |
| Sail |  |
| Gill Net |  |
| Cast/Hand Net |  |
| Trawl Net |  |
| Crab Trap |  |
| Long line |  |
| Hand line |  |
