# Supplementary material for: Do Social Networks Influence Small-Scale Fishermen’s Enforcement of Sea Tenure?
Source: PLoS One. 2015 Mar 30;10(3):e0121431. doi: 10.1371/journal.pone.0121431 (PMC4379162; doi:10.1371/journal.pone.0121431)
Supplement: S4 Table — (DOCX) [file pone.0121431.s004.docx]

**S4 Table.** Survey questions used to assess enforcement behavior amongst fishermen in four communities around Pearl Lagoon, Nicaragua.

| 8. In the past year, have you seen outsiders fishing in Pearl Lagoon? | | | | | | | | | | | | |
| --- | --- | --- | --- | --- | --- | --- | --- | --- | --- | --- | --- | --- |
|  | | Y / N *(If no, skip to Q11*) | | | | | | | | | |  |
| 9. In the past year, have you gone directly to talk to an outsider who was fishing in your area? | | | | | | | | | | | |  |
|  | | Y / N *(If no, skip to Q11)* | | | | | | | | | |  |
| 9a. When was the last time you talked to an outsider directly (month)? | | | | | | | | | | | |  |
| 9b. Why did you go talk to the person/people? | | | | | | | | | | | | |
| 9c. Who else was there when you went to go talk to the outsiders? | | | | | | | | | |  | |  |
|  | |  | |  | |  | |  | |  | |  |
| 9d. Did this time or any other time in the past year result in physical attack with outsiders? | | | | | | | | | | | | |
|  | Y / N | |  | |  | |  | |  | |  | |
| 10. In the past year, how many times did you go talk to outsiders about why they were fishing here? | | | | | | | | | | | | |
|  | A. 1 time | | | |  | |  | |  | |  | |
|  | B. 2 - 4 times | | | |  | |  | |  | |  | |
|  | C. 5 - 8 times | | | |  | |  | |  | |  | |
|  | D. More than 8 times | | | | | | | |  | |  | |
| 11. In the past year, have you requested your community leader to do something about people from outside coming to fish here? | | | | | | | | | | | | |
|  | Y / N | |  | |  | |  | |  | |  | |
| 12. In the past year, have you requested a member of the communal board to do something about people from outside coming to fish here? | | | | | | | | | | | | |
|  | Y / N | |  | |  | |  | |  | |  | |
| 13. In the past year, have you requested anyone at the muncipality to do something about people from outside coming to fish here? | | | | | | | | | | | | |
|  | Y / N | |  | |  | |  | |  | |  | |
| 14. In the past year what else have you done to try to stop outsiders from fishing in Pearl Lagoon? | | | | | | | | | | | | |
|  |  | |  | |  | |  | |  | |  | |
